# Aztreonam-avibactam Demonstrates Potent Activity Against Carbapenem-resistant Enterobacterales Collected From US Medical Centers Over a 6-year Period (2017–2022)

**DOI:** 10.1093/ofid/ofaf250

**Published:** 2025-04-25

**Authors:** Krisztina M Papp-Wallace, Helio S Sader, Joshua M Maher, John H Kimbrough, Mariana Castanheira

**Affiliations:** JMI Laboratories/Element-Iowa City, North Liberty, Iowa, USA; JMI Laboratories/Element-Iowa City, North Liberty, Iowa, USA; JMI Laboratories/Element-Iowa City, North Liberty, Iowa, USA; JMI Laboratories/Element-Iowa City, North Liberty, Iowa, USA; JMI Laboratories/Element-Iowa City, North Liberty, Iowa, USA

**Keywords:** antimicrobial resistance, avibactam, aztreonam, carbapenem-resistant Enterobacterales, β-lactam-β-lactamase-inhibitor combinations

## Abstract

**Background:**

Carbapenem-resistant Enterobacterales (CREs) are a major public health threat because treatment options are limited. The predominant resistance mechanism in CREs is the production of carbapenemases. Aztreonam-avibactam was shown to possess activity against CREs with class A, B, and D carbapenemases. Herein, the activity of aztreonam-avibactam and comparators were evaluated against Enterobacterales collected between 2017 and 2022 in the United States.

**Methods:**

Antimicrobial susceptibility testing for aztreonam-avibactam, ceftazidime-avibactam, imipenem-relebactam, meropenem-vaborbactam, cefiderocol, tigecycline, and colistin was conducted using the reference broth microdilution method. Isolates resistant to imipenem or meropenem were defined as carbapenem-resistant, underwent whole-genome sequencing, and were analyzed for the presence of carbapenemase genes.

**Results:**

Of the 54 576 Enterobacterales, 0.9% were CREs and only 3.7% of the CREs were susceptible to aztreonam; the addition of avibactam increased susceptibility to 98.4% based on EUCAST breakpoints. Cefiderocol and tigecycline were the next most potent agents, inhibiting 94.7% of the CREs at current breakpoints. Whole-genome sequencing analysis of the CREs revealed that 82.6% carried a carbapenemase with 360 isolates having only a class A carbapenemase. KPC-encoding genes were the predominant carbapenemase genes identified with 50.4% found in CREs from New York, New Jersey, and Pennsylvania. Aztreonam-avibactam was highly active against CREs carrying class A, B, and/or D carbapenemases with susceptibility rates of 99.4%, 98.0%, and 100%, respectively. Moreover, 94.4% of isolates with no carbapenemases detected were susceptible to aztreonam-avibactam.

**Conclusions:**

Aztreonam-avibactam demonstrates potent activity toward CREs with different carbapenem-resistance mechanisms. The combination is an anticipated welcome addition to the clinician's toolbox giving physicians another option to treat CREs.

## Teaser

The activity of aztreonam-avibactam was evaluated against a contemporary collection of carbapenem-resistant Enterobacterales from the United States. Of the 511 carbapenem-resistant Enterobacterales tested, 98.4% were susceptible to aztreonam-avibactam. The predominant underlying carbapenem-resistance mechanism identified was the presence of *bla*_KPC_.

The Centers for Disease Control and Prevention define carbapenem-resistant Enterobacterales (CRE) as an urgent threat to public health [1]. Because some CREs are resistant to almost all clinically available antibiotics, the Centers for Disease Control and Prevention recommends that aggressive action be taken to combat this group of pathogens. The current guidance from the Infectious Diseases Society of America for the treatment of CREs varies based on infection source, severity, and the phenotype of the CREs, with β-lactam agents being the preferred agents over tigecycline and colistin [2]. Carbapenem-resistance in Enterobacterales is predominantly conferred via the production of β-lactamases that hydrolyze carbapenems (ie, carbapenemases) and are referred to as carbapenemase-producing Enterobacterales (CPEs) [3]. Four classes (ie, class A, B, C, and D) of β-lactamases exist and are categorized based on their primary amino acid sequence, overall structure, as well as mechanism of action. The catalytic mechanism of action of class A, C, and D β-lactamases differs significantly from class B enzymes, which are metallo-β-lactamases. Carbapenemases are found within 3 of these classes, class A (eg, KPCs), class B (eg, VIM, NDM, IMP), and class D (OXA-48-like enzymes).

Identifying a single inhibitor that targets all β-lactamases with different hydrolysis mechanisms is a significant scientific challenge. Modern clinically available β-lactam/β-lactamase inhibitor combinations, ceftazidime-avibactam, meropenem-vaborbactam, and imipenem-relebactam possess some activity against CPEs. However, their activity is inadequate against CPEs producing class B carbapenemases and is variable against CPEs with class D carbapenemases. Thus, there is a critical medical need for the continued development of agents with activity against these pathogens.

Aztreonam-avibactam was shown to possess antimicrobial activity against CPEs regardless of the carbapenemases present [4–8]. The utility of this combination is attributable to aztreonam being a poor substrate for class B carbapenemases [9, 10] and class D carbapenemases [11]. Concomitantly, avibactam acts as potent inactivator of class A and class D OXA-48–like carbapenemases as well as narrow and extended-spectrum class A and C β-lactamases [12]. Thus, the combination of aztreonam and avibactam provides a unique opportunity to target all CPEs regardless of the β-lactamases produced [4]. In April 2024, aztreonam-avibactam as a single-dose vial containing 1.5 g of aztreonam and 0.5 g of avibactam was approved for use in the European Union by the European Medicines Agency for the treatment of adult patients with complicated intra-abdominal infections, hospital-acquired pneumonia, including ventilator-associated pneumonia, and complicated urinary tract infections, including pyelonephritis. It is also indicated for the treatment of infections due to aerobic Gram-negative organisms in adult patients with limited treatment options. In the United States, aztreonam-avibactam is currently in clinical development for the same infection types. In this study, the activity of aztreonam-avibactam and comparators was evaluated against >500 CRE isolates, including both CPEs and non-CPEs collected in US hospitals during a 6-year period (2017–2022).

## METHODS

### Organism Collection

From 2017 to 2022, a total of 54 576 Enterobacterales isolates were collected from patients across the United States from 62 medical centers that participated in the INFORM Program for all 6 years. Isolates were obtained from patients with urinary tract infections, skin/soft-tissue infections, nosocomial pneumonia, bloodstream infections, and intraabdominal infections. Isolates were limited to 1 per patient. CREs included *Citrobacter amalonaticus/farmeri* (1), *C freundii species complex* (23), *C koseri* (2), *Enterobacter cloacae species complex* (82), *Escherichia coli* (29), *E vulneris* (1), *Hafnia alvei* (1), *Klebsiella aerogenes* (31), *K oxytoca* (31), *K pneumoniae* (264), *Proteus mirabilis* (2), *Providencia rettgeri* (3), *Raoultella ornithinolytica* (1), *Serratia marcescens* (34), and unspeciated *Raoultella* (6). Isolates were identified using standard biochemical tests, MALDI Biotyper (Bruker Daltonics; Billerica, MA), or whole-genome sequencing (WGS) as needed.

### Susceptibility Testing

Isolates were subjected to susceptibility testing against aztreonam-avibactam, ceftazidime-avibactam, imipenem-relebactam, meropenem-vaborbactam, cefiderocol, tigecycline, and colistin using the reference broth microdilution method as described by the Clinical and Laboratory Standards Institute (CLSI) [13]. Avibactam and relebactam were tested at a fixed concentration of 4 mg/L. Vaborbactam was tested at a fixed concentration of 8 mg/L. Cefiderocol powder was acquired from MedChem Express (Monmouth Junction, NJ) and tested using iron-depleted media. Quality control was performed according to the CLSI [14]. All quality control minimum inhibitory concentration (MIC) results were within acceptable ranges. Categorical interpretations for all comparator agents, except tigecycline, were those criteria found in the CLSI M100 (2022) [14]. For tigecycline, the categorial interpretations of the US Food and Drug Administration's Antibacterial Susceptibility Test Interpretive Criteria website was used because it is not available in the CLSI M100. Categorial interpretations for aztreonam-avibactam were based on the EUCAST (2024) breakpoints of S ≤ 4 and R > 4 as CLSI and FDA breakpoints are not available.

### Whole-genome Sequencing

CRE isolates resistant to imipenem and/or meropenem underwent WGS and data analysis for the detection of β-lactamase genes, as previously described [15, 16]. Briefly, WGS was performed on a MiSeq or NextSeq (Illumina, San Diego, CA, USA) instrument targeting a 30× coverage. Sequences were de novo assembled. Analysis of β-lactam resistance mechanisms was performed in silico. Resistance genes were searched using a curated library and a criterion of >94% sequencing identity and 40% minimum length coverage was applied.

### Characterization of Aztreonam-avibactam–resistant Isolates

\Isolates with aztreonam-avibactam Minimum Inhibitory Concentration (MIC) values >4 mg/L (resistant, EUCAST) were further genetically characterized through extraction of sequences encoding acquired and intrinsic resistance genes (eg, carbapenemases, chromosomal *ampC* genes). Additionally, the amino acids sequences of penicillin-binding protein 3 (FtsI) and the porins OmpC/OmpF (*E coli* and *S marcescens*) and the homologous OmpK36/OmpK35 (*K aerogenes, K oxytoca*) were compared to the corresponding sequences from the susceptible reference isolates *E coli* ATCC 25922, *K aerogenes* KCTC2190 (PBP3)/ATCC 13048 (OmpK36)/OmpK35 (ATCC 15038), and *K oxytoca* KCTC1686.

Gene expression of *acrA*, *ompC/ompK36,* and *ompF/ompK35* were assessed. Isolates were inoculated into fresh cation adjusted Mueller-Hinton broth to 0.5 McFarland units (Thermo Fisher Scientific; Waltham, MA, USA), diluted 1:10 in fresh tryptic soy broth, and grown to log phase (optical diameter_600_ 0.3–0.5). Cells were then mixed 1:2 with RNA Protect, incubated 5 minutes at room temperature, and centrifuged for 10 minutes at 3700 relative centrifugal force before RNA extraction using the RNA Easy Mini kit (Qiagen; Hilden, Germany) on a Qiacube workstation (Qiagen). Total RNA was treated with RQ1 RNase-free DNase (Promega; Madison, WI, USA) and cleaned using the RNA Easy Mini kit; RNA integrity was assessed using the Agilent RNA 6000 pico kit (Agilent Technologies; Santa Clara, CA, USA). Gene expression for each target (Supplementary Table 1) was determined in triplicate for each sample with 0.5 ng of RNA per reaction using the Power SYBR Green RNA-to-C_T_ 1-step kit on a StepOnePlus quantitative polymerase chain reaction workstation (Applied Biosystems-Thermo Fisher Scientific). Gene expression was normalized to an internal housekeeping gene (*gyrA, rpsL,* or 16S) and compared to a susceptible control strain using the StepOnePlus analysis platform (Applied Biosystems).

## RESULTS

### Aztreonam-avibactam Demonstrates Potent Activity Against Enterobacterales, Including CREs

The MIC_50_ and MIC_90_ values for aztreonam for all the 54 576 Enterobacterales isolates collected in the United States between 2017 and 2022 were 0.12 mg/L and >16 mg/L, respectively. The addition of avibactam decreased the aztreonam MIC_50_ and MIC_90_ values to ≤0.03 mg/L and 0.12 mg/L, respectively. MIC_50_ and MIC_90_ values over time remained stable with only a slight uptick for the MIC_90_ value of aztreonam (16 mg/L vs > 16 mg/L) that occurred between 2019 and 2020.

Of the 54 576 Enterobacterales, 0.9% were CREs, accounting for a total of 511 isolates. The addition of avibactam to aztreonam shifted the MIC profiles toward the susceptible range with only 3.7% of isolates susceptible to aztreonam alone to 98.4% susceptible when avibactam was added using the aztreonam-avibactam EUCAST breakpoint (Table 1 and Supplementary Figure 1) [17]. Aztreonam-avibactam was more active than any of the other agents tested including cefiderocol and tigecycline. The rank order of potency against CREs was aztreonam-avibactam > cefiderocol = tigecycline > ceftazidime-avibactam > meropenem-vaborbactam > imipenem-relebactam, with the corresponding susceptibility rates of 98.4% > 94.7% = 94.7% > 89.2% > 86.7% > 81.6%, respectively (Table 1).

**Table 1.** Activity of Antimicrobial Agents Tested Against 511 CRE Isolates Collected in the United States From 2017 to 2022^a,b^

Agent	MIC_50_ (mg/L)	MIC_90_ (mg/L)	Range (mg/L)	%S	%I	%R
Aztreonam	>16	>16	≤0.03–>16	3.7	1.6	94.7
Aztreonam-avibactam	0.25	1	≤0.03–>16	98.4	N/A	1.6
Cefiderocol	1	4	≤0.004–>64	94.7	2.5	2.7
Tigecycline	0.5	2	≤0.06–8	94.7	4.3	1.0
Colistin	0.25	>8	≤0.06–>8	N/A	84.7	15.3
Ceftazidime	>32	>32	0.12–>32	5.9	2.5	91.6
Ceftazidime-avibactam	1	32	≤0.015–>32	89.2	N/A	10.8
Meropenem	8	>32	0.06–>32	3.7	10.2	86.1
Meropenem-vaborbactam	0.06	16	≤0.015–>32	86.7	2.7	10.6
Imipenem	8	>8	0.5–>8	4.1	3.5	92.4
Imipenem-relebactam	0.25	8	0.03–>8	81.6	2.9	15.5

Abbreviations: CLSI, Clinical and Laboratory Standards Institute; CRE, carbapenem-resistant Enterobacterales; FDA, Food and Drug Administration; MIC, minimum inhibitory concentration; N/A, not applicable.

^a^Criteria as published by CLSI (2022) was used for interpretation of all agents with exception of tigecycline where US FDA (2022) breakpoints were used, and for aztreonam-avibactam MICs, EUCAST breakpoints of S ≤ 4 and R > 4 were used.

^b^CLSI M100 standard is recognized.

### *bla*_KPC_ Remains the Dominant Carbapenemase Gene in US Isolates

Analysis of the WGS of the 511 CREs revealed that 82.6% of isolates carried a carbapenemase (Table 2). The most common carbapenemase was *bla*_KPC_ at 68.7% followed by *bla*_NDM_ at 8.6% and *bla*_OXA-48-like_ at 3.1%; *bla*_SME_, *bla*_NMC-A_, *bla*_IMP_, and *bla*_VIM-1_ were also identified, but were rarer (Table 2 and Supplementary Figure 2*A*). *bla*_KPC-2_ and *bla*_KPC-3_ were the sole carbapenemase genes found in 145 and 196 isolates, respectively.

**Table 2.** Occurrence of Carbapenemases Detected Among 511 CRE Isolates Collected During 2017–2022 in US Hospitals

Carbapenemases	No. of Isolates	% of CRE
Class A serine-carbapenemases	360	70.5
KPC-2	145	28.6
KPC-3	193	37.8
KPC-4	3	0.6
KPC-6	2	0.4
KPC-6, KPC-10	1	0.2
KPC-18	1	0.2
KPC-58	1	0.2
KPC-59	1	0.2
KPC-195	1	0.2
NMC-A	2	0.4
SME-2	5	1.0
SME-4	5	1.0
Class B MBLs	50	9.8
NDM-1	24	4.7
NDM-1, KPC-3	1	0.2
NDM-1, OXA-181	1	0.2
NDM-1, OXA-232	1	0.2
NDM-5	14	2.7
NDM-5, KPC-3	1	0.2
NDM-5, OXA-181	2	0.4
VIM-1	2	0.4
IMP-4	2	0.4
IMP-4, KPC-3	1	0.2
IMP-27	1	0.2
Class D OXA-48-like β-lactamases	12	2.3
OXA-48	7	1.4
OXA-181	2	0.4
OXA-232	3	0.6
Carbapenemase negative	89	17.4
Total	511	…

Abbreviations: CRE, carbapenem-resistant Enterobacterales; MBL, metallo-β-lactamase.

Assessing prevalence of carbapenemases over time, the number of *bla*_KPC_ genes present in Enterobacterales declined while the number of metallo-β-lactamase genes slightly increased over time (Supplementary Figure 2*A*). Of the 50 strains carrying metallo-β-lactamases, 7 isolates also carried a second carbapenemase, either a class A *bla*_KPC-3_ or a class D *bla*_OXA-48-like_ gene (Table 2). Other CPEs were not found to carry multiple carbapenemases with one exception of an isolate with 2 *bla*_KPC_ genes (*bla*_KPC-6_ and *bla*_KPC-10_).

### CREs are Most Prevalent in the Middle Atlantic States of New Jersey, New York, and Pennsylvania

CREs were most commonly isolated in the Middle Atlantic (ie, New Jersey, New York, and Pennsylvania) with 44.4% of all CREs coming from this region of the US (Supplementary Figure 2*B*). Of the 227 CREs from the Middle Atlantic, 177 carried a class A serine carbapenemase, 29 had a metallo-β-lactamases, and 18 were carbapenemase negative. The next region most prevalent for CREs is West South Central (ie, Texas, Oklahoma, Arkansas, and Louisiana) at 13.9% with 49, 3, 5, and 15 isolates with class A carbapenemases, metallo-β-lactamases, class D carbapenemases, and no carbapenemases, respectively. New England had the fewest CREs with only 7 isolates. Metallo-β-lactamases were found in isolates from every region except West North Central (ie, North Dakota, South Dakota, Nebraska, Kansas, Minnesota, Iowa, and Missouri).

### Aztreonam-avibactam is Highly Active Against CPEs as Well as Non-CPEs

The activity of aztreonam-avibactam was compared to clinically available β-lactam-β-lactamase inhibitor combinations of ceftazidime-avibactam, meropenem-vaborbactam, and imipenem-relebactam. Against CREs carrying only class A carbapenemases, the activities of the different available β-lactam-β-lactamase inhibitor combinations were comparable with 92.8%–99.4% isolates being susceptible (Supplementary Figure 3 and Table 3). Conversely, aztreonam-avibactam displays the greatest antimicrobial activity against CREs producing class B carbapenemases with 98.0% of isolates testing susceptible. For CREs with class D carbapenemases, aztreonam-avibactam and ceftazidime-avibactam were both highly active, with 100% of isolates susceptible to both agents (Supplementary Figure 3*B* and 3*C*). Of the CREs not producing carbapenemases, only 5.6% of 89 isolates were resistant aztreonam-avibactam, moreover other β-lactam-β-lactamase inhibitor combinations demonstrated similar activity with only 2.2%–5.6% of non-CPE CREs being resistant to these agents.

**Table 3.** Activity of β-lactam-β-lactamase Combinations, Cefiderocol, Tigecycline, and Colistin Tested Against 511 CRE Isolates Separated by β-lactamase Class and Presence or Absence^a,b^

Agent	MIC_50_ (mg/L)	MIC_90_ (mg/L)	Range (mg/L)	%S	%I	%R
CREs with only class A carbapenemases (360 isolates)						
Aztreonam-avibactam	0.25	0.5	≤0.03–8	99.4	N/A	0.6
Ceftazidime-avibactam	1	2	<0.03–32	98.9	N/A	1.1
Meropenem-vaborbactam	0.03	0.5	<0.03–>32	98.3	0.6	1.1
Imipenem-relebactam	0.125	1	0.03–>8	92.8	2.2	5.0
Cefiderocol	0.5	2	<0.03–64	98.9	0.3	0.8
Tigecycline	0.5	2	≤0.06–8	96.7	1.9	1.4
Colistin	0.25	>8	≤0.06–>8	N/A	82.7	17.3
CREs with class B carbapenemases^c^ (50 isolates)						
Aztreonam-avibactam	0.25	0.5	≤0.03–8	98.0	N/A	2.0
Ceftazidime-avibactam	>32	>32	0.125–>32	4.0	N/A	96.0
Meropenem-vaborbactam	16	>32	0.5–>32	16.0	6.0	78.0
Imipenem-relebactam	8	>8	1–>8	2.0	4.0	94.0
Cefiderocol	2	16	<0.03–32	74.0	14.0	12.0
Tigecycline	0.5	2	0.25–4	92.0	8.0	0.0
Colistin	0.25	16	0.06–>8	N/A	86.0	14.0
CREs with only class D carbapenemases (12 isolates)						
Aztreonam-avibactam	0.25	0.5	≤0.03–0.5	100.0	N/A	0.0
Ceftazidime-avibactam	1	2	0.125–2	100.0	N/A	0.0
Meropenem-vaborbactam	32	>32	1–>32	16.7	8.3	75.0
Imipenem-relebactam	4	8	1–>8	16.7	0.0	83.3
Cefiderocol	0.5	2	<0.03–4	100.0	0.0	0.0
Tigecycline	1	4	0.25–4	83.3	16.7	0.0
Colistin	0.125	0.25	≤0.06–>8	N/A	91.7	8.3
CREs without carbapenemases (89 isolates)						
Aztreonam-avibactam	0.5	4	0.03–>16	94.4	N/A	5.6
Ceftazidime-avibactam	1	8	<0.03–>32	96.6	N/A	3.4
Meropenem-vaborbactam	1	8	0.03–16	88.8	9.0	2.2
Imipenem-relebactam	0.25	2	0.06–4	89.9	5.6	4.5
Cefiderocol	1	8	0.03–64	88.8	5.6	5.6
Tigecycline	0.5	4	≤0.06–4	89.9	10.1	0.0
Colistin	0.25	2	≤0.06–>8	N/A	91.0	9.0

Abbreviations: CRE, carbapenem-resistant Enterobacterales; MIC, minimum inhibitory concentration; N/A, not applicable.

^a^Criteria as published by CLSI (2022) was used for interpretation of all agents with exception of tigecycline where US FDA (2022) breakpoints were used, and for aztreonam-avibactam MICs, EUCAST breakpoints of S ≤ 4 and R > 4 were used.

^b^CLSI M100 standard is recognized.

^c^Includes 7 isolates with *bla*_KPC-3_ (3) or *bla*_OXA-48_-like (4) genes.

Contrasting the activity of aztreonam-avibactam with last resort agents (eg, tigecycline, colistin) and cefiderocol used to treat infections caused by CREs, carbapenemase type was associated with varied susceptibility profiles (Table 3). Cefiderocol and tigecycline were comparable to aztreonam-avibactam against CREs with class A carbapenemases with 0.6%–1.4% of isolates testing resistant to these agents (Table 3). On the contrary, 17.3% of CREs with class A carbapenemases were resistant to colistin (Table 3). Against CREs with class B carbapenemases, tigecycline was the second most active agent next to aztreonam-avibactam with 92.0% of isolates susceptible, whereas only 74.0% were susceptible to cefiderocol (Table 3). Cefiderocol, tigecycline, and colistin demonstrated similar potency against CREs with class D carbapenemase as well as those with no carbapenemases (Table 3).

### Isolates Resistant to Aztreonam-avibactam Possess Combinations of Mechanisms

Eight isolates, *E coli* (3), *K aerogenes* (2), *K oxytoca* (1), *S marcescens* (1), and *E cloacae* species complex (1) possessed MIC results that were ≥8 mg/L to aztreonam-avibactam (Supplementary Table 2). Four of these 8 isolates were non-CPEs that were carbapenem resistant. Five, 3, and 4 of the 8 isolates were also nonsusceptible to ceftazidime-avibactam, meropenem-vaborbactam, and imipenem-relebactam, respectively. Two of the aztreonam-avibactam–resistant isolates were also resistant to cefiderocol, whereas 1 was resistant to colistin, but all were susceptible to tigecycline.

The isolates underwent further molecular characterization including identification of acquired and intrinsic β-lactam–resistance genes, amino acid sequence analysis of β-lactamases, penicillin-binding protein 3 (FtsI), porins (OmpC/OmpF in *E coli* and *S marcescens* and OmpK36/OmpK35 in *K aerogenes* and *K oxytoca*), as well as expression analysis of porins and the *acrA* gene that encodes an efflux pump. Amino acid insertions/substitutions in PBP3 and porins and decreased expression of porins likely contributed to the elevated aztreonam-avibactam MICs in these strains (Supplementary Table 2).

## CONCLUSIONS

Aztreonam-avibactam displayed potent antimicrobial activity against CREs collected between 2017–2022 in the United States. Only 1.6% of the 511 CRE isolates were resistant to aztreonam-avibactam based on EUCAST breakpoints. Of the 1.6%, 50% were non-CPE CREs, thus they lacked a carbapenemase, which implies that nonenzymatic-mediated mechanism of carbapenem resistance (eg, porin loss) also impacts aztreonam-avibactam. Modern clinically available β-lactam-β-lactamase inhibitor combinations that are recommended CRE treatment options showed good activity with 81.6%–89.2% of isolates testing susceptible. Moreover, ∼5% of the CREs were nonsusceptible to cefiderocol or tigecycline. All CREs were whole-genome sequenced and interrogated for the presence of carbapenemase genes. The *bla*_KPC_ gene was the most prevalent carbapenemase; thus, it continues to be the most prevalent carbapenemase in the United States [18]. Compared to a previous study [8], in which a subset of these CREs were evaluated, a downtrend in *bla*_KPC_ prevalence continued; however, in contrast, class B and class D carbapenemase prevalence slightly decreased from 2021 to 2022, but overall the prevalence of both remain higher compared to previous years. Overall, the activity of the agents tested herein varies depending on the β-lactamases present

## Supplementary Material

ofaf250_Supplementary_Data

## Supplementary Data

Supplementary materials are available at *Open Forum Infectious Diseases* online. Consisting of data provided by the authors to benefit the reader, the posted materials are not copyedited and are the sole responsibility of the authors, so questions or comments should be addressed to the corresponding author.

## Notes

***Acknowledgments.*** The authors thank the participant sites of the INFORM Program for the providing isolates.

***Author Contributions*.** K.M.P.-W.: formal analysis, writing—original draft; J.M.M: formal analysis, investigation, methodology; J.K.: formal analysis, investigation, methodology; H.S.S.: conceptualization, formal analysis, funding acquisition, investigation, project administration, resources, supervision; M.C.: conceptualization, formal analysis, funding acquisition, investigation, project administration, resources, supervision.

***Financial support.*** This study was performed by Element Iowa City (JMI Laboratories) and supported by AbbVie Inc., which included funding for services related to preparing this manuscript.

***Transparency statement*.** Element Iowa City (JMI Laboratories) was contracted to perform services in 2023 for AbbVie Inc., Adamed Pharma S.A., AimMax Therapeutics, Allecra Therapeutics, Amicrobe, Inc., AN2 Therapeutics, Inc., Apnimed, Astellas Pharma, Inc., Basilea Pharmaceutica AG, Baxis Pharmaceuticals, Inc., Beckman Counter, Inc., bioMérieux, Biosergen AB, Blacksmith Medicines, Bugworks, Carnegie Mellon University, Center for Discovery and Innovation, Cerba Research NV, Cidara Therapeutics, Cipla USA Inc., ContraFect Corporation, CorMedix Inc., Crestone, Inc., Discoveric Bio Beta Ltd., Dr. Falk Pharma Gmbh, Entasis Therapeutics, Evopoint Biosciences, Fedora Pharmaceuticals, Forge Therapeutics, GARDP Foundation, Genentech, Gilead Sciences, Inc., GlaxoSmithKline plc, Harvard University, Institute for Clinical Pharmacodynamics, Iterum Therapeutics plc, Janssen Biopharma, Johnson & Johnson, Kbio, Inc., Lakewood-Amedex Inc, Locus Biosciences, Inc., McGill University, Medpace, Inc, Meiji Seika Pharma, Melinta Therapeutics, Menarini Group, Merck & Co., MetCura Pharmaceuticals, Inc., MicuRx Pharmaceutical Inc., Mundipharma International Ltd., Nabriva Therapeutics, National Cancer Institute, National Institutes of Health, NovoBiotic Pharmaceuticals, LLC, Ohio State University, Omnix Medical Ltd., Paratek Pharmaceuticals, Pfizer, Inc, PPD Global Central Labs, LLC, Pulmocide Ltd, Qpex Biopharma, Inc, Revagenix, Inc, Roche Holding AG, Scynexis, Inc., Seed Health, SeLux Diagnostics, Shionogi & Co., Ltd., Spero Therapeutics, Sumitovant Biopharma, Inc., Swedish National Reference Laboratory, TenNor Therapeutics, ThermoFisher Scientific, U.S. Food and Drug Administration, University of Wisconsin, VenatoRx Pharmaceuticals, Wockhardt, and Zoetis, Inc.
