# Supplementary material for: Aztreonam-avibactam Demonstrates Potent Activity Against Carbapenem-resistant Enterobacterales Collected From US Medical Centers Over a 6-year Period (2017–2022)
Source: Open Forum Infect Dis. 2025 Apr 25;12(5):ofaf250. doi: 10.1093/ofid/ofaf250 (PMC12069807; doi:10.1093/ofid/ofaf250)
Supplement: ofaf250_Supplementary_Data [file ofaf250_supplementary_data.zip › Supplemental Figure Legends final.docx]

**Supplemental Figure Legends**

**Supplemental Table 1.** Primers used for gene expression analysis

**Supplemental Table 2.** Summary of the results on the characterisation of isolates exhibiting aztreonam-avibactam MIC results ≥8 mg/L.

**Supplemental Figure 1.** Aztreonam and aztreonam-avibactam MIC distribution for 511 CREs collected from 2017-2022 from US Hospitals

**Supplemental Figure 2. A.** Prevalence of major carbapenemase families over time in CRE isolates and **B.** the distribution of CREs by US Census Divisions collected from 62 medical centers through the SENTRY surveillance program from 2017-2022 (Note: Pie charts values depict total number of carbapenemases or non-carbapenemases per region not per isolate; while the CRE number is the number of total isolates).

**Supplemental Figure 3.** Susceptibility patterns for β-lactam-β-lactamase inhibitor combinations vs. CREs carrying **A.** Class A carbapenemase, **B.** Class B carbapenemase, and **C.** Class D carbapenemases and **D.** CREs without carbapenemases.
