# Supplementary material for: Aztreonam-avibactam Demonstrates Potent Activity Against Carbapenem-resistant Enterobacterales Collected From US Medical Centers Over a 6-year Period (2017–2022)
Source: Open Forum Infect Dis. 2025 Apr 25;12(5):ofaf250. doi: 10.1093/ofid/ofaf250 (PMC12069807; doi:10.1093/ofid/ofaf250)
Supplement: ofaf250_Supplementary_Data [file ofaf250_supplementary_data.zip › Supplemental Tables.docx]

**Supplemental Table 1.** Primers used for gene expression analysis

|  | Forward^a^ | Reverse^a^ |
| --- | --- | --- |
| *E. coli* |  |  |
| *acrA* | GTTGGCGTAGTAACAGTCAA | CGGTAACTTCTTGTGCTTTT |
| *ompC* | GCTAACAACATCTACCTGG | ACAGCTTCGAAGTTCTG |
| *ompF* | TCTGTACGGTAAAGCTGTTG | CGATCTGGTTGATGATGTAG |
| *rpsL* | TACATCGGTGGTGAAGGTCA | ACTTGGAACGAGCCTGCTTA |
| *K. aerogenes* |  |  |
| *acrA* | ACAGGATGTGACGATAAACCG | TTGTAGAGGTGCGGATTTGAG |
| *ompC* | TGACTTCCAGAAACAGAACGG | TTCACCCAGAACACCGTAAC |
| *ompF* | AAACCCAGATCAACGACCAG | ATTCACCCGCTTTCAGACC |
| *rpsL* | GACTAACGGTTTCGAAGTC | ACGAACGGTGTGGTAAC |
| *K. oxytoca* |  |  |
| *acrA* | ACGACCATCGCGCTGGCATC | GCGCGTCTGGAAGAAGGCGT |
| *ompK35* | TGCTGAAGCCGTCGCCGTTT | ACGGCGTAGCCACCTACCGT |
| *ompK36* | CGTAGGTGTCGCCGCCGAAT | AGGCGTGGACTCGTCTGGCA |
| *gyrA* | ATCTGCCCGTGTCGTGGGTG | GTCGCCGTCGACCGAACCAA |
| *S. marcescens* |  |  |
| *acrA* | GCTGAATCTCGACTTTACCCG | CGAAATAGACGTAGACCTTGTCG |
| *ompC* | CTTACACCTACTCCGACAACTTC | CGCCGATATCATAAGTACAGG |
| *ompF* | TTCTTGCAGTGGTTATCCCG | GAACATAGGTCTTATCGCCGTC |
| 16S | CCCAGATGGGATTAGCTAGTAGGT | TGGCTGGTCATCCTCTCAGA |

^a^ Primers are shown 5’🡪3’

**Supplemental Table 2**. Summary of the results on the characterisation of isolates exhibiting aztreonam-avibactam MIC results ≥8 mg/L.

|  |  | | |  | |  | | | |  | | |  | |  | | |  |  |  | |  | |  |  | |  | | | Relative Expression Interpretation  (fold change) | | |
| --- | --- | --- | --- | --- | --- | --- | --- | --- | --- | --- | --- | --- | --- | --- | --- | --- | --- | --- | --- | --- | --- | --- | --- | --- | --- | --- | --- | --- | --- | --- | --- | --- |
| Organism | | Year | State | | AZA | | CZA | MEV | IMR | | FDC | TIG | | COL | | MLST | Intrinsic β-lactamase | | | | Acquired ESBL/ Carbapenemase | | PBP3 | | | OmpC/ OmpK36 | | OmpF/ OmpK35 | | *acrA* | *ompC/ ompK36* | *ompF/ ompK35* |
| *Enterobacter ludwigii* | | 2017 | NY | | 8 | | 2 | 0.5 | 8 | | 1 | 0.25 | | 0.12 | | 258 | ACT-54 | | | | NMC-A | | ND | | | ND | | ND | | ND | ND | ND |
| *Serratia marcescens* | | 2017 | NY | | 8 | | 32 | 0.5 | 2 | | 0.25 | 2 | | >8 | | N/A | SRT (Q21H, E73Q, N102K, R107H, D182G, E235K, S236P, K294Q, V372I) | | | | KPC-3 | | F221S, V562I | | | Altered | | Altered | | Baseline (0.86) | Baseline (0.47) | Baseline (0.63) |
| *Klebsiella oxytoca* | | 2019 | CA | | 8 | | 32 | 16 | 0.5 | | 0.25 | 1 | | 0.25 | | 194-like | OXY-1-2 (G75S, N173G) | | | | N/A | | V576M | | | Disrupted | | WT | Baseline (4.76) | | Baseline (0.24) | Baseline (1.76) |
| *Klebsiella aerogenes* | | 2021 | TX | | 8 | | 8 | 4 | 1 | | 2 | 0.5 | | 0.25 | | 93 | AmpC (A300V) | | | | N/A | | WT | | | Nonsense | | WT | Baseline (0.46) | | Reduced (0.01) | Reduced (0.10) |
| *Klebsiella aerogenes* | | 2021 | KY | | >16 | | >32 | 2 | 2 | | >64 | 0.5 | | 0.25 | | 176 | AmpC (G123E, Q140K, S189G, P299S, E301G, V303L, N322K, S327P, V328L, N366Y) | | | | N/A | | WT | | | Nonsense | | WT | Baseline (2.85) | | Baseline (6.8) | Baseline (0.98) |
| *Escherichia coli* | | 2021 | NY | | 8 | | >32 | >32 | 8 | | 4 | 0.25 | | 0.25 | | 405 | EC-26 (S102I, Q196H, N201T, C325R, T367A) | | | | NDM-5/  CTX-M-15 | | E149D, T233A, V332I,  P333_Y334insYRIK,  A413V | | | Disrupted | | Nonsense | Baseline (0.32) | | Baseline (2.35) | Reduced (0.0007) |
| *Escherichia coli* | | 2022 | TX | | 8 | | 16 | 8 | 0.25 | | 0.5 | 0.12 | | 0.12 | | 744 | EC | | | | N/A | | E149D, T233A,  A317T, V332I,  S408Y, A498T | | | Disrupted | | Nonsense | Moderate (7.32) | | Reduced (0.0004) | Reduced (0.005) |
| *Escherichia coli* | | 2022 | IN | | 16 | | 8 | 2 | 0.25 | | >64 | 0.25 | | 0.12 | | 405 | EC-26 (S102I, Q196H, N201T, C325R, T367A) | | | | CTX-M-33/  CTX-M-15 | | E149D, T233A,  V332I,  P333_Y334insYRIK,  A413V | | | Nonsense | | Nonsense | Baseline (0.63) | | Reduced (0.18) | Reduced (0.0008) |

^a^ Abbreviations: MIC, minimum inhibitory concentration; AZA, aztreonam-avibactam; CZA, ceftazidime-avibactam; MEV, meropenem-vaborbactam; IMR, imipenem-relebactam; FDC, cefiderocol; TIG, tigecycline; COL, colistin; MLST, multilocus sequence type; WT, wildtype; N/A, not applicable, ND, not determined.

^b^ Expression results were reported as fold changes relative to a susceptible control isolate.
