# Supplementary figures and images for: Aztreonam-avibactam Demonstrates Potent Activity Against Carbapenem-resistant Enterobacterales Collected From US Medical Centers Over a 6-year Period (2017–2022)
Source: Open Forum Infect Dis. 2025 Apr 25;12(5):ofaf250. doi: 10.1093/ofid/ofaf250 (PMC12069807; doi:10.1093/ofid/ofaf250)

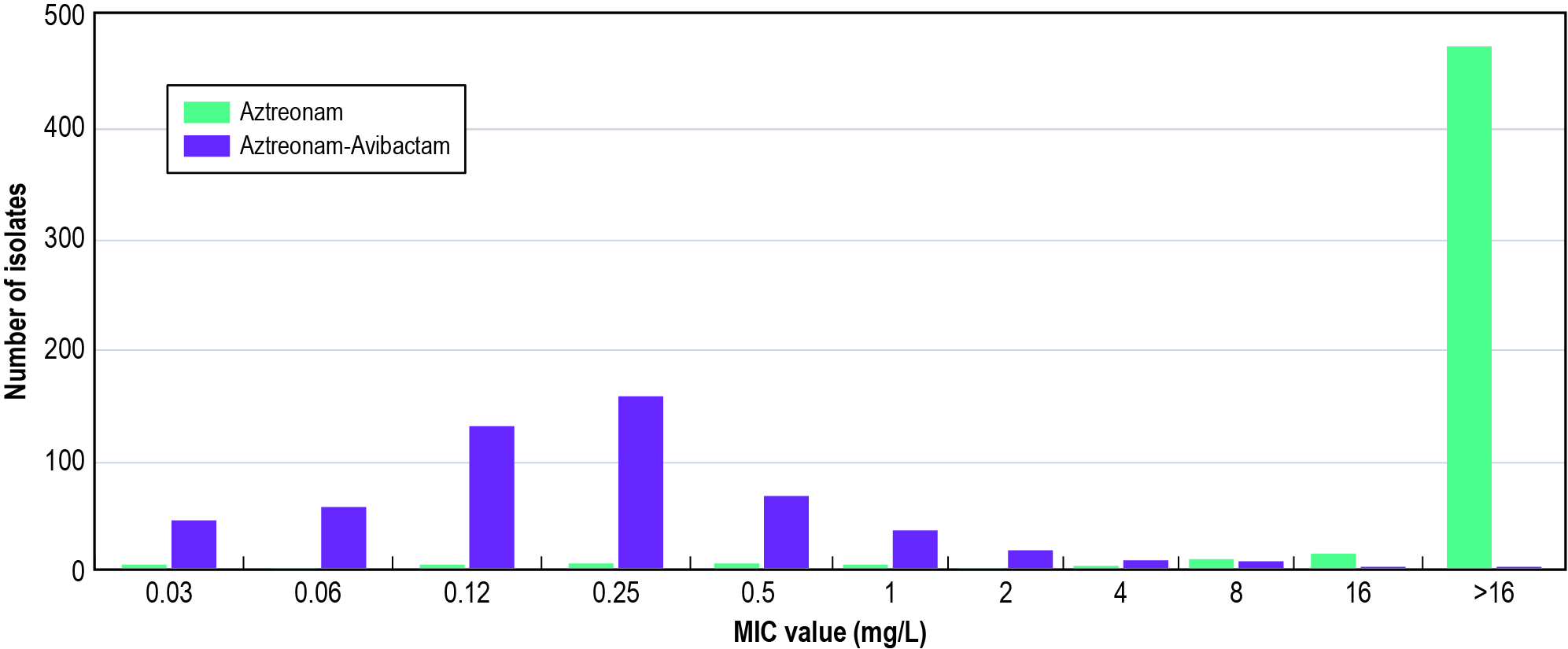

Supplement: ofaf250_Supplementary_Data [file ofaf250_supplementary_data.zip › OFID_22-ALG-04 M1_Figure1.tif]

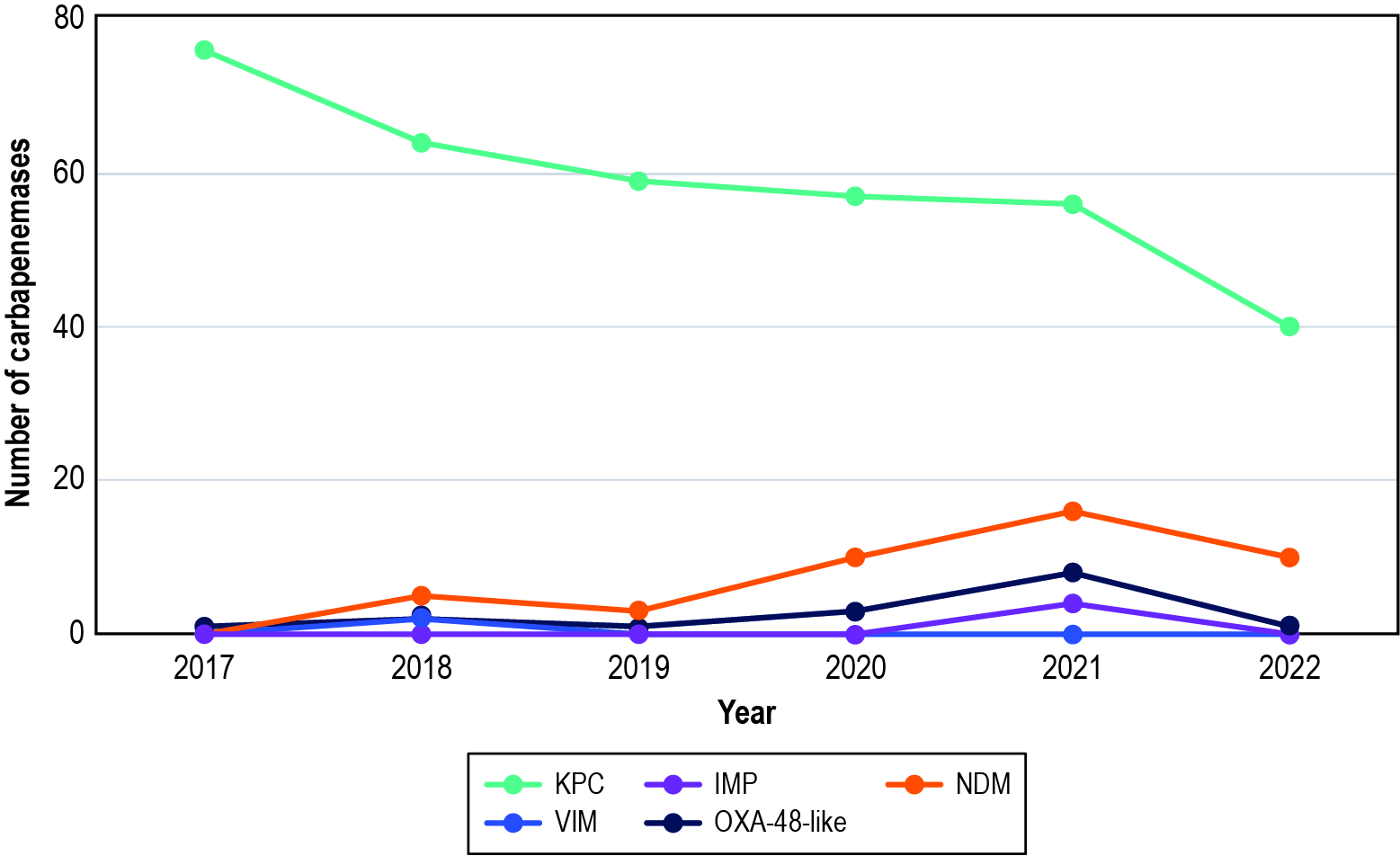

Supplement: ofaf250_Supplementary_Data [file ofaf250_supplementary_data.zip › OFID_22-ALG-04 M1_Figure2A.tif]

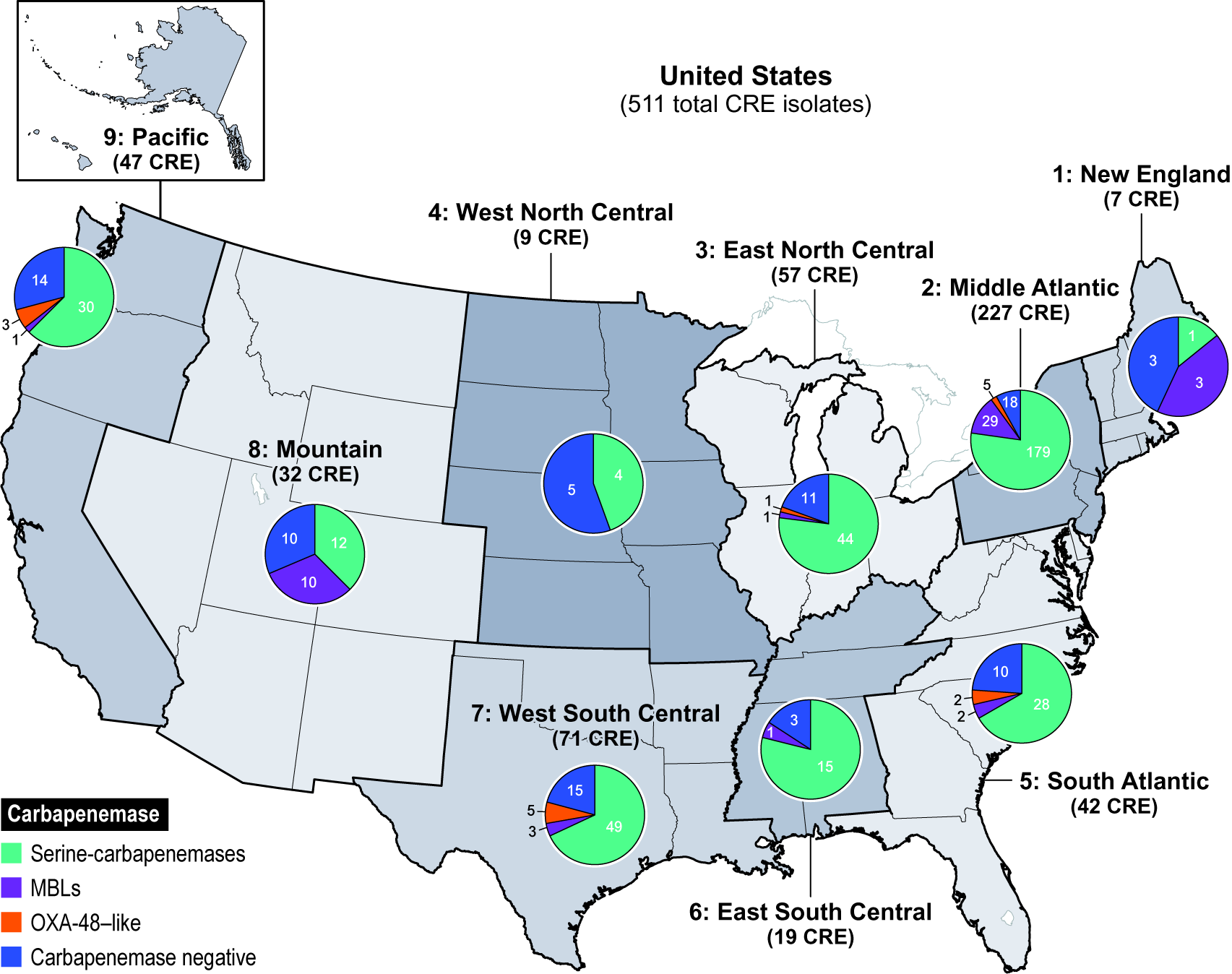

Supplement: ofaf250_Supplementary_Data [file ofaf250_supplementary_data.zip › OFID_22-ALG-04 M1_Figure2B.tif]

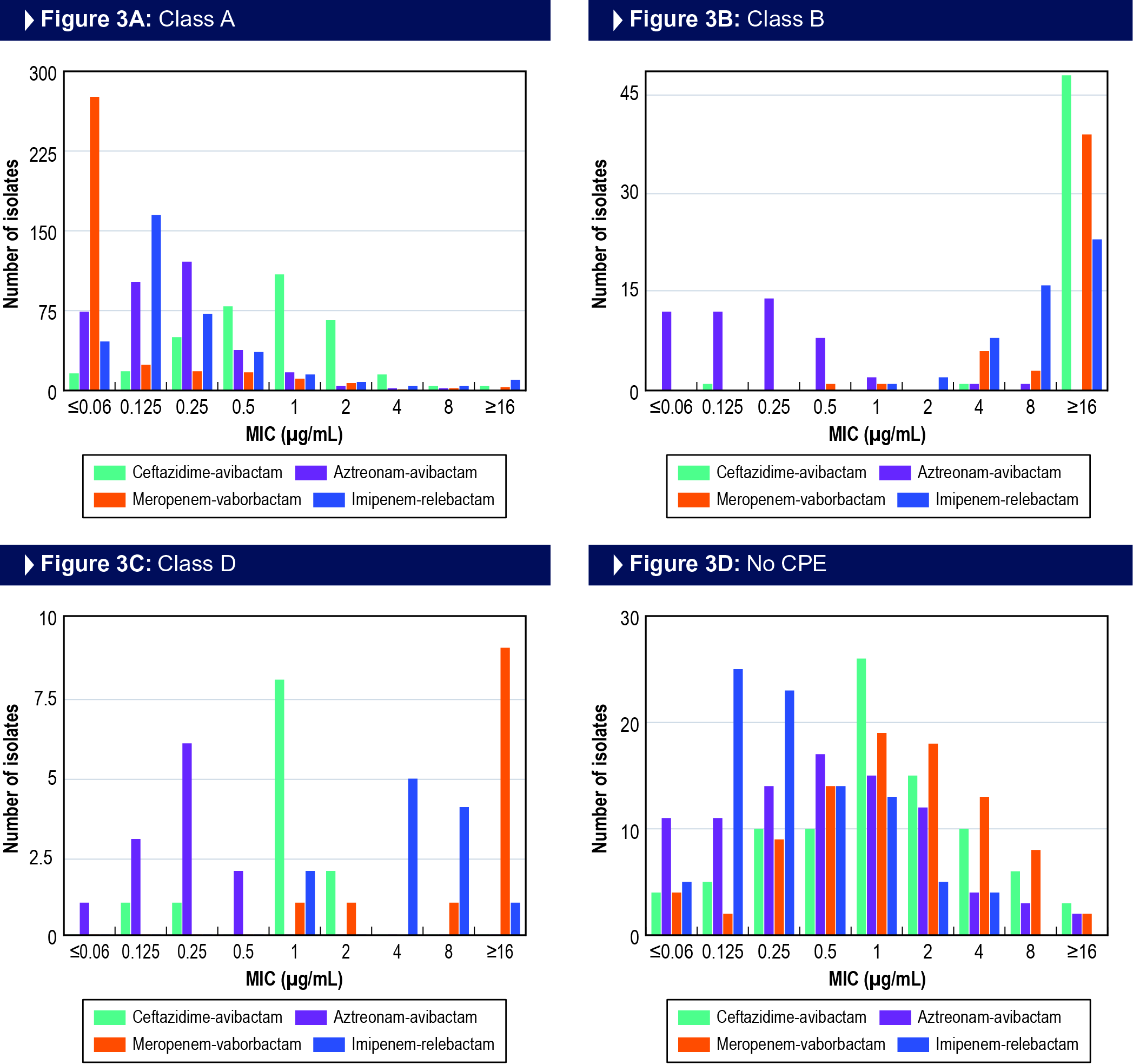

Supplement: ofaf250_Supplementary_Data [file ofaf250_supplementary_data.zip › OFID_22-ALG-04 M1_Figure3A-3D.tif]
